# Influence of Pericarp, Cotyledon and Inhibitory Substances on Sharp Tooth Oak (*Quercus aliena* var. *acuteserrata*) Germination

**DOI:** 10.1371/journal.pone.0047682

**Published:** 2012-10-25

**Authors:** Yan Liu, Guangquan Liu, Qingmei Li, Yong Liu, Longyu Hou, GuoLei Li

**Affiliations:** 1 Key Laboratory for Silviculture and Conservation of Ministry of Education, Beijing Forestry University, Beijing, China; 2 China Institute of Water Resources and Hydropower Research, Beijing, China; 3 Northwest Agriculture and Forestry University, Yangling, China; 4 State Key Laboratory of Tree Genetics and Breeding, Research Institute of Forestry, Chinese Academy of Forestry, Beijing, China; 5 State Key Laboratory of Vegetation and Environmental Change, Institute of Botany, Chinese Academy of Sciences, Beijing, China; United States Department of Agriculture, United States of America

## Abstract

In order to explore the mechanism of delayed and uneven germination in sharp tooth oak (*Quercus aliena* var. *acuteserrata*) (STO), mechanical scarification techniques were used to study STO root and shoot germination and growth. The techniques used were: removing cup scar (RS), removing the pericarp (RP), and cutting off 1/2 (HC) and 2/3 (TC) cotyledons. Germination percentage and root and shoot length for Chinese cabbage (*Beassica pekinensis*) seeds (CCS) were also investigated for CCS cultivated in a Sanyo growth cabinet watered by distilled water and 80% methanol extracts from the acorn embryo, cotyledon and pericarp with concentrations of 1.0 g, 0.8 g, 0.6 g and 0.4 g dry acorn weight per ml methanol. The results showed that the majority of roots and shoots from acorns with RP and HC treatment emerged two weeks earlier, more simultaneously, and their total emergences were more than 46% and 28% higher, respectively. TC accelerated root and shoot emergence time and root length, but root and shoot germination rate and shoot height had no significant difference from the control. Positive consequences were not observed on all indices of RS treatment. The germination rates of CCS watered by 1.0 g·ml^−1^ methanol extracts from the embryo and cotyledon were significantly lower than those from the pericarp, and all concentrations resulted in decreased growth of root and shoot. Methanol extracts from pericarp significantly reduced root length of CCS, but presented little response in germination percentage and shoot length. The inhibitory effect was gradually increased with the increasing concentration of the methanol extract. We conclude that both the mechanical restriction of the pericarp and the presence of germination inhibitors in the embryo, cotyledon and pericarp are the causes for delayed and asynchronous germination of STO acorns.

## Introduction

Oaks (*Quercus* L), the temperate-zone forest tree species and the most commercially important hardwood genus [1], [2], are widely distributed across Asia, Europe, North America and Africa. However, over several decades, numerous scientists have observed a failure in natural regeneration of oaks [3]–[7]. Therefore, in recent years, oak container nurseries have become increasingly important in oak production. Container nurseries require simultaneous seedling germination and emergence, as longer germination periods allow the earlier germinated plants to quickly develop leaves which overshadow neighboring seedlings and restrict access to water [8]. However, oaks germinate asynchronously under natural conditions and the difference between first and last germinating acorns can be up to a few weeks [9]. In addition, acorns of the subgenus *Erythrobalanus* usually exhibit delayed germination [2]. Suszka et al. [9] have indicated that in oaks, the epicotyls start to develop 20 days later than the roots. Acorns of *Quercus aliena* send up a thick tap root growing down several centimeters into the soil instead of a green shoot in the fall [10]. All of these occurrences have a negative effect on the quality of seedlings.

For researchers, improving our understanding of the reasons leading to delayed and uneven germination and finding corresponding improvement approaches can not only develop the quality of seedlings and increase the rate of natural oak regeneration, but also can have guiding significance for seedling. Over the last 20 years, researchers have summarized that four main factors are responsible for delayed and asynchronous germination: (1) mechanical strength of the pericarp [2], [11]; (2) secondary metabolites such as tannins and polyphenols [12]; (3) plant hormones, especially abscisic acid (ABA) and indoleacetic acid (IAA) affecting acorn metabolic pathways [13], [14]; and (4) germination inhibitors, which are chemicals produced in plants that prevent the germination of their own seeds or seeds of other species [15]. Blanche [16] has found inhibitory substances in aqueous extracts of pericarp tissue, which increased along with acorn development. The inhibitory substances’ presence has also been confirmed in the pericarp of mature water oak (*Quercus nigra*) acorns [17]. Following these discoveries, some corresponding measures have been developed, including physical (mechanical restraint of the pericarp and cotyledon), chemical (secondary metabolites) and physiological (abscisic acid, growth regulator and other inhibitors) mechanisms. Removing pericarp from the distal end of the acorn [2], [18] and cutting off parts of the distal ends of the acorns [1], [19] can cause faster and more uniform germination. The cotyledon reserves at the apex of acorns are more important than those at the base in supporting acorn viability and seedling establishment [1].

Sharp tooth oak is one of the most important tree species of economy and ecology in warm temperature zone and north subtropical zone of China, especially in Qingling Mountains and also in Korea and Japan. As they are rich in nutrition, acorns are often infested by predators. Some researches have indicated that those infested acorns only damaged pericarp and small cotyledon may improve seedling emergence [1], [19], [20], while others have shown negative effects [7] and some studies have kept the medium [21]. The mechanical treatment which can imitate the infestation in the natural condition is focus on cork oak (Q. variabilis) [1], [22], pedunculate oak (*Q. variabilis*) [1], [22], pedunculate oak (*Quercus robur* L.) [8], [19], water oak (*Q. nigra* ) [23] and so on. However, the similar research on sharp tooth oak acorns is rare. And the previous studies on mechanical scarification have dealt with acorn germination and seedling emergence only. The consequences of this procedure for root and seedling development are little understood [19], especially root development. The germination inhibitors of oak acorns have also been little studied.

The aim of this study was to determine the influence of removing the pericarp and cutting off part of the distal end of acorns of sharp tooth oak (STO) (*Quercus aliena var. acuteserrata*) in different degree and whether the pericarp, cotyledon and embryo of acorns had germination inhibitors. We hypothesized that removing the pericarp and a small reduction of the distal end of cotyledon would be favorable for roots and seedlings because the reduction of cotyledon reserves will be compensated by speeding up emergence. We assumed that the pericarp, cotyledon and embryo would have germination inhibitors which could lead to delayed and asynchronous germination.

## Materials and Methods

### Acorn Collection and Treatment

Acorns from STO parent trees in the Beijing Botanical Garden of the Chinese Academy of Sciences (116°20' E, 39°56' N) were collected from the ground in mid-September 2009. Acorns were immersed in water in the laboratory and all of those still floating after five minutes were removed [21], [24]. The remaining acorns were air dried and stored in 5 ml (0.127 mm) polyethylene bags in a low temperature apparatus ZPZ - 1000 (made by Hangzhou Qianjiang Apparatus & Equipment CO. LTD) at 3±1°C. Polyethylene can be permeable to carbon dioxide and oxygen, yet largely impermeable to moisture [2].

**Figure 1:**
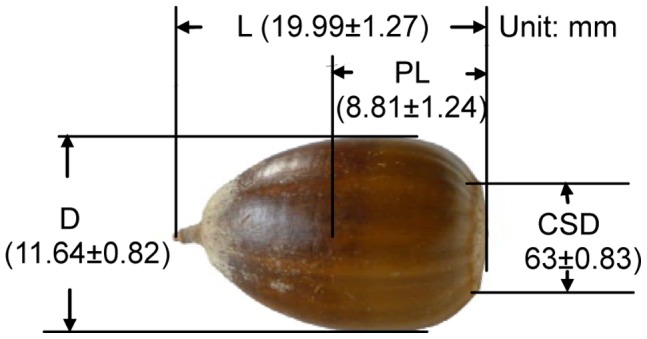
Profile of acorn measurement. L-Length, PL- Package length, D- Diameter, CSD- Cup scar diameter.

After acorns are air dried, some basic seed morphological characteristics were measured (Figure 1). Acorn mass (1.58±0.31 g) was measured by 1/1000 electronic balance and the other measures were determined using a vernier caliper.

### Acorn Germination

**Figure 2:**
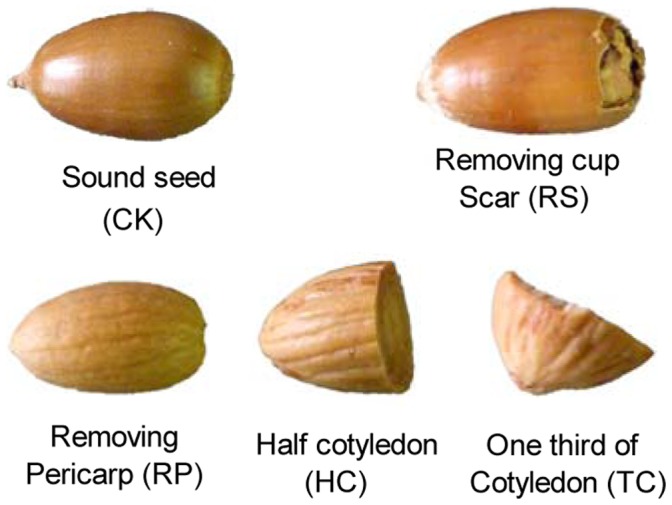
The schematic diagram of different mechanical treatments of sharp tooth oak acorn.

On September 19, 2009, acorns were tested for germination on top of three pieces of paper moistened with distilled water in 11.5 cm diameter Petri dishes at 25°C with 8 hours light in an incubator. Before testing, acorns were randomly assigned to five experimental treatments (Figure 2): 1- control (CK), 2- removing cup scar (RS), 3- removing the pericarp (RP), 4- removing the pericarp and cutting off half of the cotyledon (HC), 5- removing the pericarp and cutting off 2/3 of the cotyledon (TC). Every treatment had three replicates of 15 grains each. As acorns germinated, root and shoot emergence were noted every seven days, and the length of all roots and shoots were measured at the end of the experiment (totaling 137 days). A seed was considered to have germinated when the protruding radicel achieved the length of the longest dimension of the seed.

### Distillation of Methanol Extracts from the Embryo, Cotyledon and Pericarp of STO Acorns and Bioassay Tests

STO acorns were divided into three parts: pericarp, cotyledon (cutting off 2/3 of the distal end of acorns) and embryo (here including embryo and 1/3 cotyledon around it). Three parts were oven-dried (30°C), crushed and passed through 1-mm sieve. Subsequently, 50 g powders of three parts were homogenized in 80% methanol extracts, sealed and extracted at 3±1°C and were then mixed together every four hours to full extraction. Methanol extracts were replaced every 24 hours and extractions were conducted three times. After methanol extracts were mixed together, they were centrifuged at 3000 r·min^−1^ for 20 min. Subsequently, the concentration of supernatants was brought to 1 g dry acorn weight per ml methanol (g·ml^−1^) by vacuum concentration. Some concentrated solutions were diluted to 0.4 g·ml^−1^, 0.6 g·ml^−1^ and 0.8****g·ml^−1^, respectively. Then 3 ml concentration extracts were added separately to the filter paper in 9 cm diameter petri dishes and the solvents were evaporated completely in air, then the same volume distilled water was added to every dish and the control was given only distilled water. The germination and growth of Chinese cabbage (*Beassica pekinensis*)) seeds (CCS) cultivated on the dishes as described above were recorded to determine the effects of inhibition of different parts of STO acorns. Before the germination test, CCS seeds were immersed in water at 45°C for about 10 min, then 100 grains of seeds were placed in germinators for testing at a daily cycle at 25°C for 8 h with lights and 16 h in the dark. Germination percentage, root and shoot length were calculated after 72 h. There were 3 replications of each treatment.

### Statistical Analyses

Absolute emergence rate over the whole period was the emergence rate recorded over a seven day period. Analysis of variance (ANOVA) was used to assess the influence of different mechanical scarification on the final root and shoot emergence rate and length and different concentration of methanol extracts from extracts from embryo, cotyledon and pericarp on germination percentage, root and shoot length of CCS. The results expressed in percent were arcsin transformed for ANOVA analyses. All analyses were conducted with SPSS 11.5 and the *F* ratio was considered significant at *P* = 0.05.

## Results

### Root and Shoot Emergence and Growth Development

**Figure 3:**
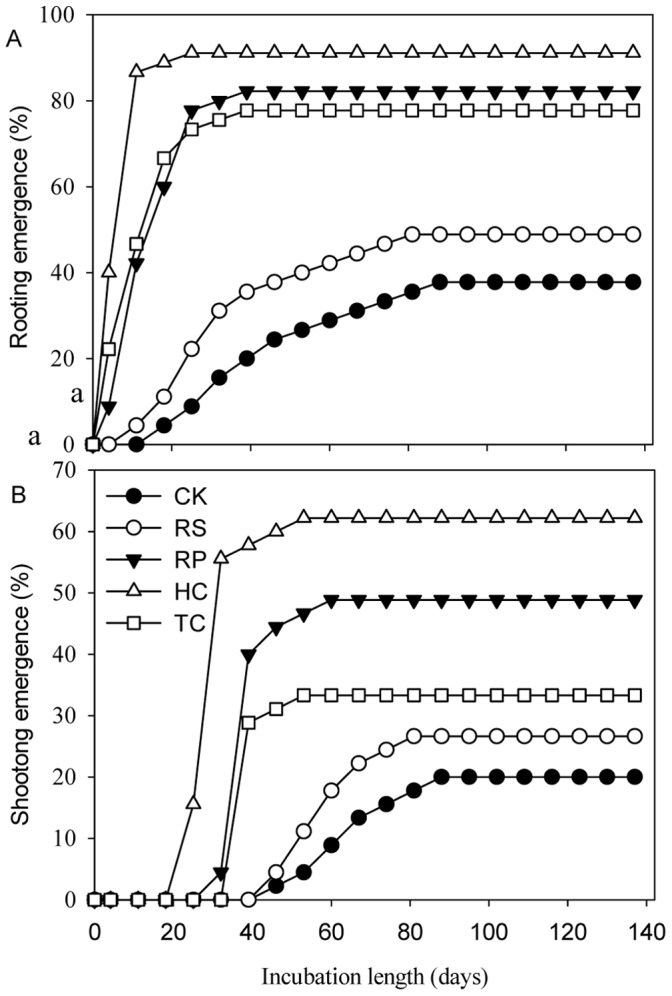
The consequent length of roots and shoots from sharp tooth oak acorns following five different mechanical scarification treatments. Data were calculated using germinant acorns to divide by all tested seeds, then multiplied by 100%. CK – the control, RS – removing the cup scar, RP – removing the pericarp, HC – removing pericarp and cutting off 1/2 of the distal end of the cotylendon, TC - removing pericarp and cutting off 2/3 of the distal end of the cotylendon.

**Figure 4:**
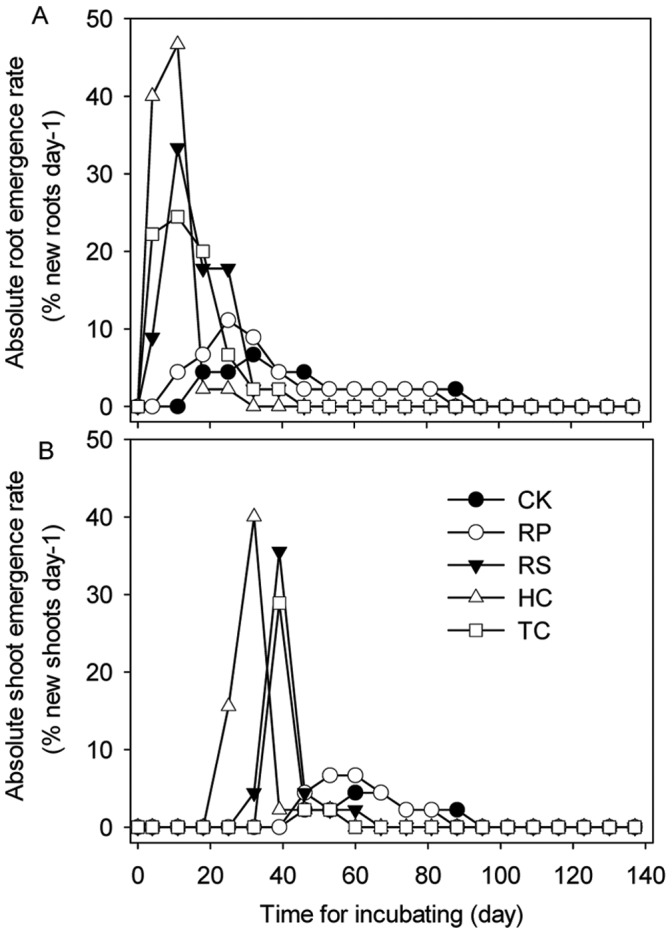
The mean absolute emergence rates of root and shoot from sharp tooth oak with five mechanical scarification treatments. Data were the emergence rate every seven days. The legend is the same as described for Figure 3 above.

The first shoots from the control treatment emerged more than 7 weeks after incubating, which delayed about 5 weeks when the first roots started emergence (Figure 3). The emergence rate of the majority roots and shoots lasted 5 and 7 weeks, respectively (Figure 4). After 137 days, 37.8% and 20.0% of control roots and shoots emerged, respectively, and the last roots and shoots started emergence even after 20 weeks (Figure 3). All these indicated that STO acorns had characteristics of delayed and irregular germination and roots without shoots.

**Figure 5:**
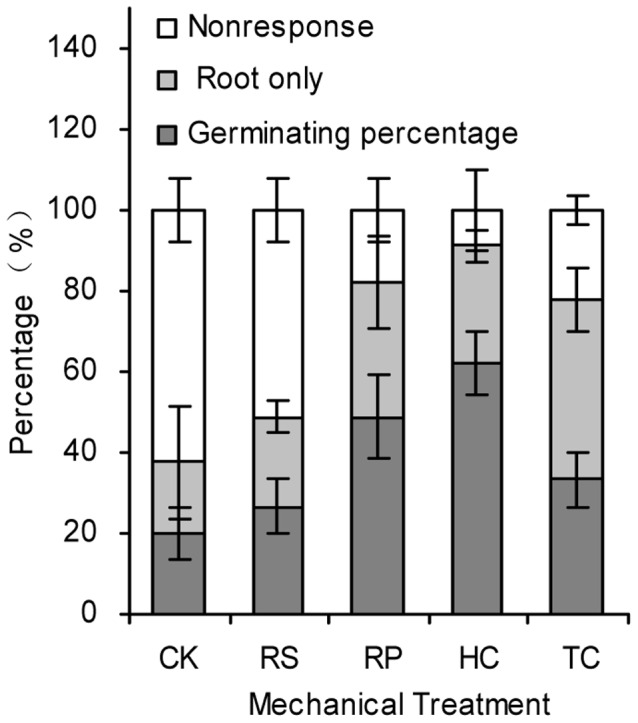
Germinating, rooted and irresponsive percentage of sharp tooth oak acorns in 137 days. Error lines represent ± standard deviation of the mean.

The reduction of acorn cotyledons caused faster and more uniform root and shoot emergence (Figure 3). The roots of RP, HC, TC treatment acorns emerged significantly (*P* = 0.016, *P* = 0.001, *P* = 0.036, respectively) better (82.2±7.7, 91.1±10.2, 77.8±3.8%, respectively) than the controls (37.8±7.7%), and the shoots from acorns with RP and HC treatment emerged significantly (*P* = 0.020, *P* = 0.001) better (48.9±10.2, 62.2±7.7%, respectively) than the controls (20.0±6.7%). The first roots from RP, HC, TC treatments emerged 4 days after sowing and the first shoots from those treatments respectively emerged 2, 3 and 1 weeks faster than those from control treatment. The majority of shoots from RP, HC, TC treatments emerged nearly simultaneously, over a two week period between 25 and 39 days after incubating (Figure 4). After 39 and 60 days, 82%, 91% and 79% root emergence and 49%, 62% and 33% shoot emergence of RP, HC, TC treatments were observed, respectively. The first roots from RS treatment started emergence 7 days earlier than those from the control treatment; however, the emergence rate of the majority of roots and shoots and final root and shoot emergence had no significant effect with the control treatment. 22%, 33%, 29% and 44% acorns from RS, RP, HC and TC treatments, respectively, had only roots but no shoots (Figure 5).

**Figure 6:**
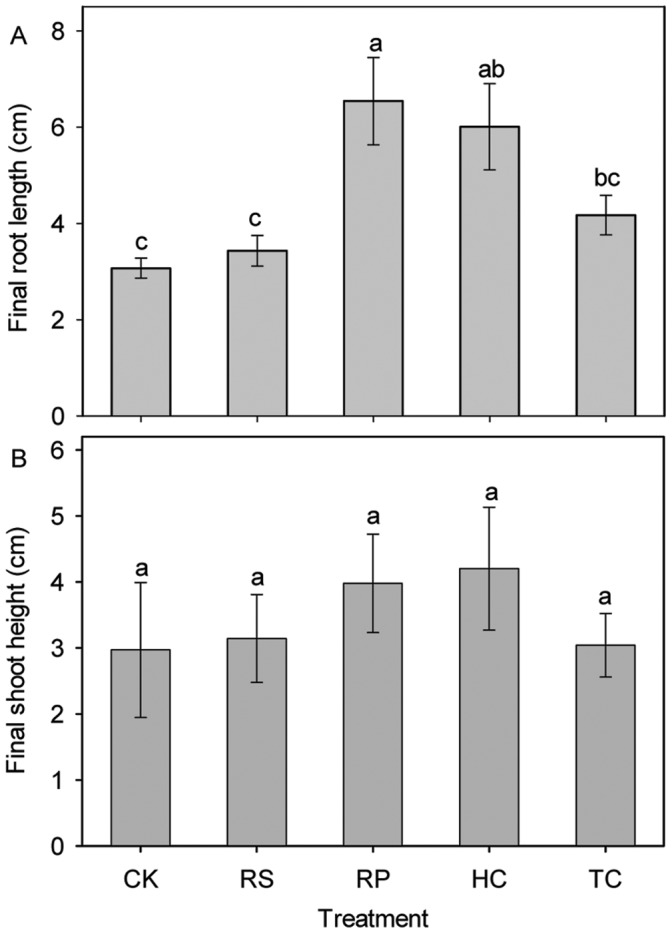
Influence of experimental treatments on mean acorn root length and seedling height (cm) at the end of the experiment. Means with the same letter are not significantly different from each other (P>0.05 ANOVA followed by Scheffe test). Error lines represent ± standard deviation of the mean. The legend is the same as described for Figure 3 above.

Final root length was significantly different between acorns across the five treatments (Figure 6A). The root lengths from RP and HC treatments were 6.5±0.91 and 6.0±0.89 cm, respectively, which were much higher than those from the control treatment (3.1±0.21 cm), while there was no significant difference between those from RS and TC and the control treatment. There was also no significant difference in shoot height between RS, RP, HC, TC and the control treatment (Fig. 6B).

### Effects of Methanol Extracts from Embryo, Cotyledon and Pericarp on CCS Germination and Growth

**Figure 7:**
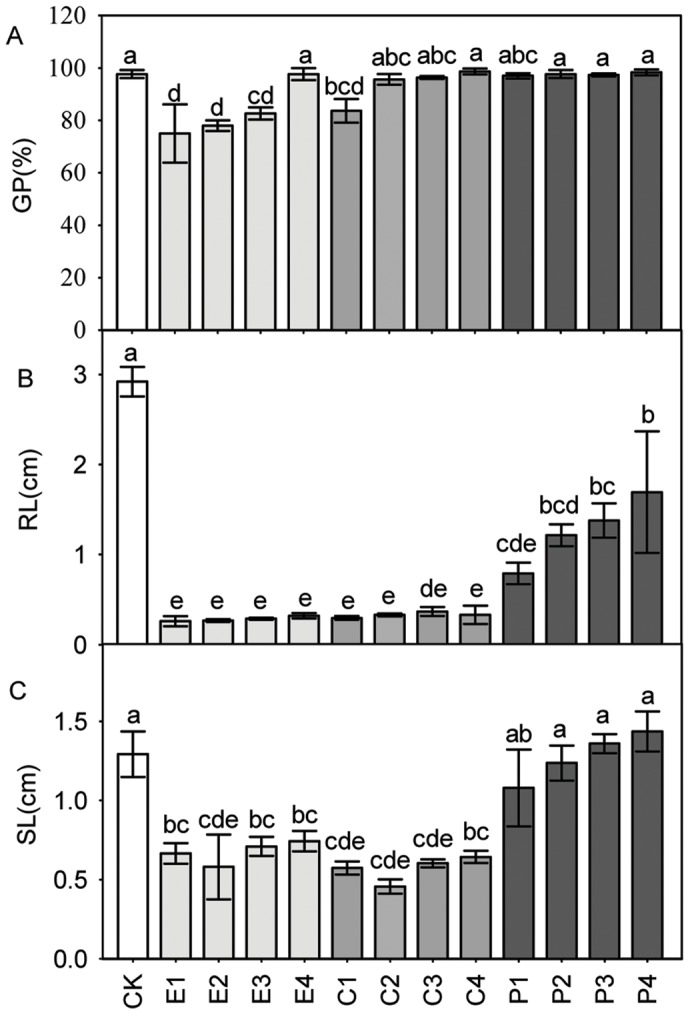
Means (±1 SD) of germination percentage (GP) (a), root length (RL) (b), and shoot length (SL) (c) for Chinese cabbage seeds. The seeds were watered by distilled water (CK) and 80% methanol extracts from the embryo (E), cotyledon(C)and pericarp (P) of sharp tooth oak acorns. E1, E2, E3 and E4 indicate extracts’ concentration of 0.4 g, 0.6 g, 0.8g and 1.0g dry acorn weight per ml methanol, respectively. Same applies for C and P. Means with the same letter are not significantly different from each other (P>0.05).

Our results clearly showed a significant difference (*P*<0.001) in germination percentage among CCS watered by distilled water (CK) and 80% methanol extracts from embryo, cotyledon and pericarp of STO acorns (Figure 7A). The germination rates of CCS watered by 1.0 g·ml^−1^, 0.8 g·ml^−1^ and 0.6 g·ml^−1^ methanol extracts from embryo and 1.0 g·ml^−1^ methanol extracts from cotyledon of STO acorns were significantly lower (*P*<0.001, *P* = 0.001, *P* = 0.014, *P* = 0.024, respectively) than CK (Figure 7 A). As the concentration of methanol extracts from embryo and cotyledon increased, the germination percentage of CCS gradually decreased. Methanol extracts by pericarp showed no significant difference in germination percentage when compared with CK.

**Figure 8:**
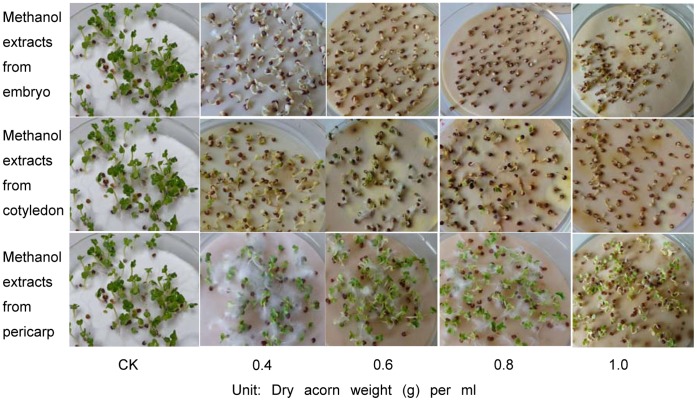
The effect picture of growth condition of Chinese cabbage seeds. The seeds were watered by distilled water (CK) and 80% methanol extracts from embryo, cotyledon and pericarp of sharp tooth oak acorns.

All concentrations of methanol extracts from embryo, cotyledon and pericarp caused significant reduction (*P*<0.001) in growth of root compared to CK (Figure 7B). The root lengths of CCS watered by embryo and cotyledon showed no significant difference, but were much shorter than those by pericarp. Shoot length also showed significant difference (*P*<0.001) among different treatments (Figure 7C). CCS watered by embryo and cotyledon had much shorter shoot length than CK and those by pericarp, while no significant difference in shoot length was detected between CCS watered by pericarp and CK (Figure 8).

## Discussion

### Effects of Pericarp on Acorn Germination

As we expected, roots and shoots from RP treatment acorns had significantly higher final root and shoot emergence and root length and emerged earlier than those from the control, whereas there was no significant difference in shoot height between RP and the control treatments. A similar result increased acorn germination from 10 to 55% but no significant effect on seedling growth has been described by Hopper and Vozzo [25] from water oak (*Q. nigra* L.) acorns. ISTA [18] has shown that removing acorn pericarp and cup scar has a positive effect on acorn emergence, while Bonner [23] has found that pericarp removal from acorns increases both emergence rate and seedling height for water oak. Previous studies have presented some reasons for higher germination rate of RP treatment acorns. According to Brown [26], the pericarp restrains cell expansion when acorns emerge, therefore a stronger force is needed for the radicel to pierce the pericarp. Hopper and Vozzo [25] have indicated that acorn is a raw material rich in tannins which can inhibit radicel emergence. The inhibitory substances’ presence has also been confirmed in the pericarp of mature water oak acorns [17]. Furthermore, the pericarp tissue might entrap gases which could affect water uptake [17], [27]. And pericarp rupture requires slow increase in capacity of acorns to imbibe the water [17], [26].

Except for faster root emergence, none of the other indices of RS treatment showed a significant difference from the control. These results were inconsistent with the work of Rakić *et al.* [12] on acorns of *Q. robur* that removing the cup scar can significantly improve the shoot germination percentage from 18% to 89%. Possible interpretations may be that (1) RS treatment reduces the mechanical restraint of the pericarp and increases water permeability to some extent, but acorns also need to break a powerful internal binding force in order to germinate; (2) RS treatment only raises the imbibing water from the acorn distal end, which still requires time for the water to transport to the radicel; and (3) the pericarp may have germination inhibitors which have a negative effect on root and shoot emergence.

### Effects of Cotyledon on Acorn Germination

Roots and shoots from reduced acorns emerged earlier than those from the control. A similar reaction from simulated acorn predation has been described by Giertych and Suszka [19] for *Q. robur*. This result has also been further proven by Suszka [8] who demonstrated that cutting off about 1/3 of the distal ends of acorns caused them to germinate faster. This is possibly due to this procedure not only allowing for easier access to external water to split the pericarp, but also improving access of water to the embryonic axis [13]. In addition, removing the cotyledon will cause development of plant growth regulators, particularly IAA – indoleacetic acid [13], [19], [28], which can also benefit radicel germination. A third explanation for faster germination following removal of the cotyledon is that cotyledons of STO readily separate when imbibed, which improves access of water to the embryonic axis [2].

The final root emergence rate of TC was significantly higher than that of the control, but no significant difference was detected in the final shoot emergence rate, root length or shoot height between TC and the control. The result was partly consistent with Andersson and Frost [29] who have indicated that cutting off cotyledons has no effect on acorn germination, but inconsistent with the work of Fukumoto and Kajimura [22] who have illustrated that cutting off too much of the cotyledon (1/2 and 1/3) has a negative effect on the growth of cork oak (*Q. variabilis*). The differences may be attributed to the cotyledon playing a different role in acorn germination among different species. Furthermore, cutting off too much cotyledon may not only reduce the acorn nutrient reserve [22], but also decrease some other essential resources in germination, as the cotyledon reserves at the apex of acorns are more important than those at the base in supporting acorn viability and seedling establishment [1].

Removing pericarp and cutting off 1/2 cotyledon, both of which can partly imitated the infestation in the natural condition, are all benefit for emergence of sharp tooth oak seeds, which was partially consistent with Branco et al [30] who have documented that cotyledon damaged by insects causes faster and more synchronous germination of *Quercus suber* acorns. Acorns with each mechanical treatment could success to germinate, well in agreement with the results of Hirka and Csóka [31], Yi and Zhang [32] who have indicated that no matter what degree the cotyledon is damaged, insect-infested acorns can still germinate to form a seedling. However, several studies have shown that infested acorns experience lower germination than the sound [33]. For acorns, both infested by insects and mechanical treatment can decrease mechanical strengthen of pericarp, and the difference is that the former usually has physiological effect while the latter has not. The different part of cotyledon infested by the predators having different effect on acorn germination may be another reason.

### Inhibitory Substance of Germination in Acorns

The germination rates of CCS watered by 1.0 g·ml^−1^ methanol extracts from the embryo and cotyledon were significantly lower than those from the pericarp, and all concentrations had a negative influence on growth of root and shoot. Methanol extracts from the pericarp significantly reduced root length of CCS, but there was little response in germination percentage and shoot length. This indicated that the embryo, cotyledon and pericarp of acorns more or less consisted of germination inhibitors which could restrain acorn germination and root or shoot length. Peterson [17] has found that the pericarp of *Q. nigra* contains inhibitor substances. Similar results were also observed in *Castanea mollissima* B I cv. ‘Yanshanhong’ and *C. mollissima* Blume by Bai *et al.* [34] and Xu *et al*. [35]. Some studies have considered that in order to defend insects, acorns can produce secondary metabolites, such as polyphenols [12] and tannins [36] which can prevent acorns germinating. Generally, the acorns of red oaks (*Erythrobalanus*) which have dormancy include more tannins than those of white oaks (*Lepidobalanus*) which have no dormancy [37]. The epicotyl dormancy of chestnut oak ( *Quercus prinus* L.) and overcup oak (*Q. lyrata* Walt.) acorns [2], [38] is one of the causes of germination inhibition.

### Meanings for Seedling Practices

By analyzing our data, the mechanical restriction of the pericarp and the germination inhibitors in three sections of acorns are responsible for delayed and uneven germination of sharp tooth oak acorns. Based on our results, both removing pericarp and cutting off 1/2 cotyledon can cause faster and simultaneous germination for sharp tooth oak acorns, and the characteristics of seedling have no significant differences with those of the control. Therefore, when sowing, the both methods above are better choices for nursery workers.
